# THE IMPACT OF INDIVIDUAL AND NEIGHBORHOOD SES ON A SMOKING CESSATION INTERVENTION IN A LUNG CANCER SCREENING SETTING

**DOI:** 10.1093/geroni/igae098.4088

**Published:** 2024-12-31

**Authors:** Jaqueline Avila, Efren Flores, Yan-Jhu Su, Jennifer Haas, Elyse Park, Nancy Rigotti

**Affiliations:** University of Massachusetts Boston, Boston, Massachusetts, United States; Massachusetts General Hospital, Boston, Massachusetts, United States; University of Massachusetts Boston, Boston, Massachusetts, United States; Massachusetts General Hospital, Boston, Massachusetts, United States; Massachusetts General Hospital, Boston, Massachusetts, United States; Massachusetts General Hospital, Boston, Massachusetts, United States

## Abstract

Lung cancer screening (LCS) offers a teachable moment for smoking cessation, but socioeconomic status (SES) may affect treatment success. This study assesses whether educational level or neighborhood SES are associated with smoking abstinence and completion of a smoking cessation intervention offered in the LCS context. This is a secondary analysis of a clinical trial (NCT03611881) testing the effectiveness of smoking cessation treatment for older smokers scheduled for LCS (N=615). Outcomes were self-reported 7-day smoking abstinence and study follow-up completion at 6-months. Independent variables were educational level (high school graduation or less [low] vs. post-high school education [high]); neighborhood SES (Area Deprivation Index [ADI], ranging 1 [low SES] to 100 [high SES] categorized as: highest 15% vs. remaining 85% scores), and a combination of both measures. Logistic regression models tested the association between outcomes and SES measures, adjusted for covariates. 32.7% of the sample had low educational level, and the mean sample ADI was 19.9 (SD: 12.8). Smoking cessation was higher among those with higher vs. lower neighborhood SES (15.7% vs. 7.4%, p-value=0.03). Study completion was lower among those with lower vs. higher educational level (78.1% vs. 84.5%, p=0.05). In multivariable models, these associations were not significant but individuals with high educational level and low neighborhood SES were more likely to complete the study than those with both low educational level and low neighborhood SES (OR: 6.04, 95%CI: 1.47-24.7). Smoking cessation in the LCS context might be improved by targeting community factors that affect neighborhood SES and educational level.

